# Racial/ethnic differences in pre-pregnancy conditions and adverse maternal outcomes in the nuMoM2b cohort: A population-based cohort study

**DOI:** 10.1371/journal.pone.0306206

**Published:** 2024-08-12

**Authors:** Meghan E. Meredith, Lauren N. Steimle, Kaitlyn K. Stanhope, Marissa H. Platner, Sheree L. Boulet

**Affiliations:** 1 H. Milton Stewart School of Industrial and Systems Engineering, Georgia Institute of Technology, Atlanta, Georgia, United States of America; 2 Department of Gynecology & Obstetrics, Emory University, Atlanta, Georgia, United States of America; Universita degli Studi dell’Insubria, ITALY

## Abstract

**Objectives:**

To determine how pre-existing conditions contribute to racial disparities in adverse maternal outcomes and incorporate these conditions into models to improve risk prediction for racial minority subgroups.

**Study design:**

We used data from the “Nulliparous Pregnancy Outcomes Study: Monitoring Mothers-to-be (nuMoM2b)" observational cohort study. We defined multimorbidity as the co-occurrence of two or more pre-pregnancy conditions. The primary outcomes of interest were severe preeclampsia, postpartum readmission, and blood transfusion during pregnancy or up to 14 days postpartum. We used weighted Poisson regression with robust variance to estimate adjusted risk ratios and 95% confidence intervals, and we used mediation analysis to evaluate the contribution of the combined effects of pre-pregnancy conditions to racial/ethnic disparities. We also evaluated the predictive performance of our regression models by racial subgroup using the area under the receiver operating characteristic curve (AUC) metric.

**Results:**

In the nuMoM2b cohort (n = 8729), accounting for pre-existing conditions attenuated the association between non-Hispanic Black race/ethnicity and risk of severe preeclampsia. Cardiovascular and kidney conditions were associated with risk for severe preeclampsia among all women (aRR, 1.77; CI, 1.61–1.96, and aRR, 1.27; CI, 1.03–1.56 respectively). The mediation analysis results were not statistically significant; however, cardiovascular conditions explained 36.6% of the association between non-Hispanic Black race/ethnicity and severe preeclampsia (p = 0.07). The addition of pre-pregnancy conditions increased model performance for the prediction of severe preeclampsia.

**Conclusions:**

Pre-existing conditions may explain some of the association between non-Hispanic Black race/ethnicity and severe preeclampsia. Specific pre-pregnancy conditions were associated with adverse maternal outcomes and the incorporation of comorbidities improved the performance of most risk prediction models.

## Introduction

The United States’ maternal mortality rate, 32.9 per 100,000 live births, has worsened over the past 20 years, [1, 2] and there are staggering racial and ethnic disparities in maternal mortality [3]. Non-Hispanic Black women are three to four times more likely than their non-Hispanic White peers to die due to pregnancy [4]. Furthermore, an estimated 80% of pregnancy-related deaths are considered preventable, [5] and individuals with adverse maternal outcomes have similar preventable factors including provider failure to identify high-risk status and inappropriate management [6]. Two potential targets for addressing pregnancy-related mortality and morbidity are improvements in identifying high-risk patients and managing complicated pregnancies, which may disproportionally affect racial and ethnic minority individuals.

Factors contributing to the increasing rates of maternal mortality and morbidity in the United States are multifaceted, but one contributing factor is chronic health conditions among women of reproductive age. Cardiovascular disease is the leading cause of maternal mortality in high-income countries [7]. Specific cardiovascular diseases including hypertension, heart failure, arrhythmia, and congenital defects are associated with increased risk of adverse maternal outcomes [8]. Rising rates of congenital heart disease, diabetes mellitus, pre-pregnancy obesity, and hypertension are primary contributors driving obstetric intensive care unit admissions [9]. The risk factors for preeclampsia, a hypertensive disorder and severe adverse maternal outcome, include pre-pregnancy cardiovascular diseases, hematologic diseases (e.g. sickle cell disease), endocrine diseases (e.g. diabetes), kidney disease, autoimmune diseases, and obesity [10, 11].

Pregnant individuals with multiple co-occurring conditions, or *multimorbidity*, have even higher rates of severe maternal morbidity and postpartum readmission [12]. While multimorbidity affects a substantial and likely growing proportion of the global adult population, [13] obstetric research and practice remain largely focused on the impact of single conditions on maternal outcomes, [14] with the exception of a few studies using administrative data, [15, 16] and those creating comorbidity-based risk screening tools [17–19]. When multimorbidity is accounted for, it is typically represented as a binary indication of each condition or a count of the number of conditions. However, studies of multimorbidity have noted the existence of particular patterns of chronic conditions associated with adverse outcomes. A study by Johnson et al. (2023) found that pregnant individuals with co-occurring anemia and pregnancy-induced hypertension encountered higher rates of preeclampsia and maternal complications compared to individuals with pregnancy-induced hypertension alone [20]. A better understanding of the effects of co-occurring pre-pregnancy conditions (e.g. hematological and cardiovascular) may be important to improve clinical management and mitigate racial disparities.

Racial and ethnic minority pregnant and postpartum women experience significantly higher rates of maternal morbidity and mortality and have a higher prevalence of chronic conditions and multimorbidity compared with non-Hispanic white individuals [21–23]. Racial and ethnic minority women, particularly Black women, are more likely to develop chronic conditions at earlier ages, more likely to have complications and mortality from chronic conditions, and less likely to have their conditions adequately managed [24]. Increased rates of chronic conditions are linked to the excess burden of structural stressors across the life course experienced by minoritized racial and ethnic groups, including adverse childhood events, poverty, and racism [25–27]. Another study by Alshakhs et al. (2022) found that African Americans had the highest prevalence of multimorbidity even after factoring in age and weight class, and African Americans presented with the most distinct disease composition patterns of multimorbidity at earlier ages [28]. For example, middle-aged African American patients without obesity most prevalently presented with a distinct multimorbidity pattern consisting of an endocrine and mental disease [28]. These studies may suggest that differences in the prevalence and management of chronic condition and multimorbidity patterns across racial/ethnic groups may contribute to the existing racial/ethnic disparities in maternal outcomes.

In this study, we investigate the impact of pre-pregnancy conditions and their combined effects (multimorbidity patterns) on adverse maternal outcomes, and whether these effects mediate the relationship between racial disparities and adverse maternal outcomes in a cohort of nulliparous individuals in the United States. A better understanding of which conditions and combinations thereof drive increased maternal risk could inform the development of new clinical care standards for pregnant people with multimorbidity and potentially improve the ability to risk-stratify pregnant people early in pregnancy.

## Materials and methods

### Study population

We conducted a secondary analysis of data from the Nulliparous Pregnancy Outcomes Study: Monitoring Mothers-to-Be (nuMoM2b) prospective cohort [29, 30]. The study recruited pregnant women who would be delivering for the first time from hospitals affiliated with eight clinical centers and collected data on each participant over the course of four study visits during their pregnancy via in-clinic interviews, self-administered questionnaires, clinical measurements, and chart abstractions. The cohort included 9,289 women who enrolled and had their first study visit in the first 6 to 14 weeks of their pregnancy and who consented to the release of their data. All local institutional review boards approved the study protocol, and participants provided written informed consent prior to enrollment. The Georgia Institute of Technology Internal Review Board and the Eunice Kennedy Shriver National Institute of Child Health and Human Development both approved this secondary analysis.

We accessed the nuMoM2b data on December 8, 2021. We did not have access to information that could identify individual participants during or after data collection.

### Data preparation

**Fig 1:**
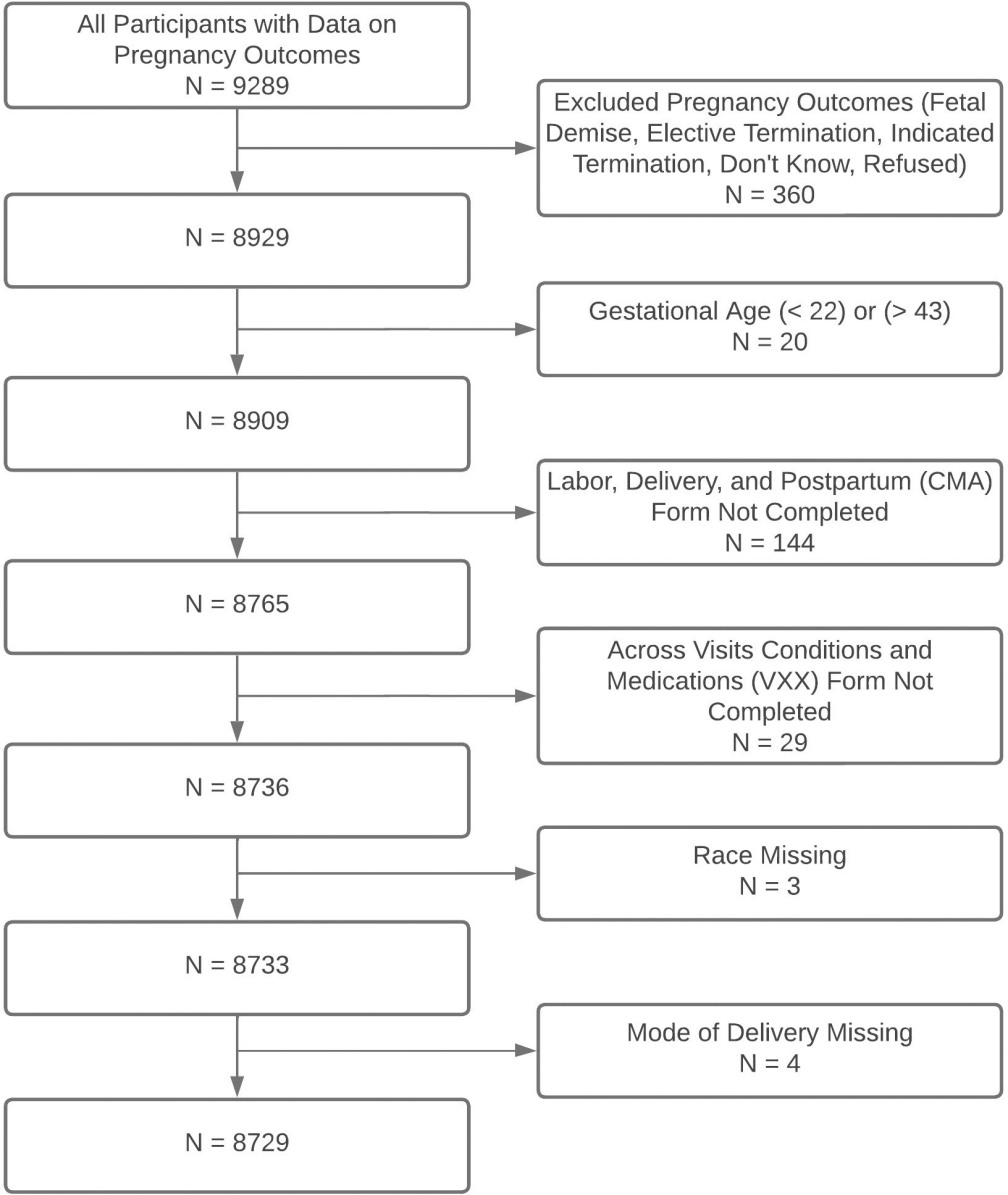
Exclusion criteria.

For this secondary data analysis, we chose to exclude women that delivered at gestational age < 22 weeks or > 43 weeks, those with terminations, and those with unknown outcomes. We also excluded women with missing race/ethnicity and pre-pregnancy conditions data (Fig 1). To address missing data among confounders, we created 5 imputed datasets using multivariate imputation by chained equations (MICE) [31]. Please see S1 Appendix for more details about the exclusion protocol and S2 Appendix for more details about the data structure and preparation.

### Main outcome measures

The primary outcomes of this study were severe preeclampsia, blood transfusion, and postpartum readmission (up to 14 days). The outcome of severe preeclampsia is indicated by one of the following diagnoses: preeclampsia with severe features, HELLP syndrome, superimposed preeclampsia, or eclampsia. The nuMoM2b definitions for these new onset hypertensive disorders are based on clinical criteria can be found in S2 Appendix of Facco et al. (2017) [32]. We selected these indicators due to their strong association with severe maternal morbidity as defined by the Centers for Disease Control and Prevention, the quality of measurement in the nuMoM2b dataset, and their impact on acute and long-term maternal health. Severe preeclampsia is a hypertensive disorder of pregnancy often presenting as new-onset hypertension and proteinuria [10]. Severe preeclampsia is a major cause of maternal morbidity, [33] and can rapidly progress to serious complications, including death of both mother and fetus [10]. Blood transfusion is included as an indicator of severe maternal morbidity (SMM) by the Centers for Disease Control and Prevention (CDC) [34]. Postpartum readmission represents a costly, rare, and heterogeneous postpartum adverse event. There is large variation in the indications for postpartum readmission in the U.S. The three most frequent indications are hypertensive disorders, postdelivery infections, and psychiatric disease [35]. (S2 Tables 1 and 2 in S2 Appendix) provides details about the definitions and collection of the primary outcomes in the nuMoM2b study. We fit regression models to each of these outcomes individually rather than create a composite outcome to preserve clinical interpretability.

### Pre-pregnancy conditions

Pre-pregnancy conditions were collected by the nuMoM2b study team at each of the first three visits through an in-person interview with the participant, who indicated the presence of each condition anytime throughout their life. The nuMoM2b study team reconciled this data with chart abstraction to finalize the indication of pre-pregnancy conditions.

The nuMoM2b study team collected 41 pre-pregnancy conditions, and we categorized them into 12 condition types according to the framework of Tang et al. [36] (S2 Table 3 in S2 Appendix). We grouped the pre-pregnancy conditions based on similarities in treatments, clinical manifestation, or organization in the health care system. For example, we grouped cervical dysplasia, fibroids, and PCOS under the condition type “Gynecological” because they are conditions that affect the function of female reproductive organs and are treated by the same gynecologist specialty branch. The condition types included: Autoimmune, Cardiovascular, Endocrine, Gastrointestinal, Gynecological, Hematologic, Kidney, Lung, Mental, and Neurological. This classification was developed and validated by three of our authors (KS, SB, MP) with extensive medical and specifically obstetric knowledge.

To investigate the effects of specific multimorbidity patterns on adverse maternal outcomes and racial/ethnic disparities, our analysis considered pairs of co-occurring pre-pregnancy condition types. For example, consider a patient with the following three pre-pregnancy conditions: anemia (hematologic), hypertension (cardiovascular), and migraine headaches (neurological). This patient was indicated to have the following co-occurring condition types: hematologic & cardiovascular, cardiovascular & neurological, and hematologic & neurological.

### Confounders

We adjusted for confounding factors including maternal age, insurance, body mass index (BMI), and sociodemographic information including income and education. Each of these factors were collected during Visit 1, which occurred between gestational age 6 and 14 weeks. Mode of delivery and type of labor were not included as confounders as they are considered to be part of the causal pathway leading to adverse maternal outcomes [37].

### Statistical analysis

We used Poisson regression models with robust standard errors to analyze the associations between pre-pregnancy condition types, self-reported race/ethnicity, and adverse maternal outcomes with the results reported as crude and adjusted risk ratios (RRs) and 95% confidence intervals (CIs). We used a class-weighting method which gives a higher priority to correctly classifying the subgroup of women who experienced an adverse maternal outcome (see S2 Appendix).

Potential mediators were selected for each adverse maternal outcome in a stepwise process to avoid multicollinearity. Each adverse maternal outcome was regressed by race/ethnicity, confounders, and all potential mediators (all pre-pregnancy condition types and all their combined effects) (hereafter referred to as the “Outcome Model”). Only condition types and combined effects reaching statistical significance (p-value ≤ 0.05) in this model were included in further analysis. Additionally, only combined effects with at least 1% prevalence in the dataset were considered. Next, we used a model-building approach starting with a standard individual-level Poisson model (Model 1, crude), followed by a Poisson model that adjusted for confounders (Model 2, adjusted). We additionally controlled for significant pre-pregnancy condition types (Model 3), and finally, we additionally controlled for significant combined effects between co-occurring pre-pregnancy condition types (Model 4).

Next, we sought to determine if pre-pregnancy condition types and their combined effects contributed to the racial disparities in adverse maternal outcomes using mediation analysis. To analyze mediation, the potential mediator condition types and their combined effects were individually regressed by race/ethnicity and adjusted for confounders (“Mediator Model”). The Outcome and Mediator models were combined to compute the mediation proportion, which estimates the proportion of the risk factor’s impact (race/ethnicity) on the outcome that is attributable to the mediator (pre-pregnancy condition type or combined effect) [38]. This analysis was conducted for each adverse maternal outcome and results were averaged across the five imputed datasets.

### Performance analysis

Finally, we analyzed the predictive performance of our regression models (Model 1, Model 2, Model 3, and Model 4) to understand which pre-pregnancy patient characteristics accurately classified adverse maternal outcomes. Performance of each model was evaluated for each racial/ethnic subgroup using the average area under the ROC curve (AUC) across 10-fold cross validation and 95% confidence intervals were computed with 2000 stratified bootstrap replicates [39]. Similar modeling was performed by Khanna et al. (2019) in which regression models were used to predict hospitalization using risk factors including the co-occurrence of two factors [40]. They report both the risk factors’ associations with hospitalization and the regression model’s predictive performance (AUC).

We used R, version 4.2.1, for all analysis, *caret* in R to train and test our prediction models, [41] *pROC* in R to compute performance metrics, [39] and *mediation* in R to conduct mediation analysis [42]. We use p-value threshold of 0.05 for statistical significance.

## Results

After exclusions, the final study population included 8729 women (Table 1). Of those, 61.0% (n = 5322) identified as non-Hispanic white, 17.0% (n = 1487) Hispanic, 13.1% (n = 1145) non-Hispanic Black, 4.0% (n = 348) multiracial, 3.9% (n = 342) Asian, and 1.0% (n = 85) “Other”. The non-Hispanic Black subgroup had the youngest average age (23.3 ± 5.3), the largest average BMI (29.1 ± 8.2), and the largest proportion with an income of less than $50,000 (43.7%). In total, the study population included 157 cases (1.8%) of blood transfusion, 154 cases (1.8%) of postpartum readmission, and 318 cases (3.6%) of severe preeclampsia. The incidence of these adverse maternal outcomes varied by race and ethnicity (S2 Table 2 in S2 Appendix). The incidence of blood transfusion and severe preeclampsia was highest in non-Hispanic Black women and the incidence of all adverse maternal outcomes was lowest in Asian women.

**Table 1:** Descriptive statistics of the nuMoM2b study population by race/ethnicity status (before imputing missing data). BMI, body mass index; Std, standard deviation; HS, high school.

		Asian	Hispanic	Multiracial	Non-Hispanic Black	Non-Hispanic White	Other
		N (%)	N (%)	N (%)	N (%)	N (%)	N (%)
Total		342 (3.9%)	1487 (17.0%)	348 (4.0%)	1145 (13.1%)	5322 (61.0%)	85 (1.0%)
**Education**							
	Degree work beyond college	158 (46.2%)	148 (10.0%)	56 (16.1%)	73 (6.4%)	1546 (29.1%)	23 (27.1%)
	Completed college	110 (32.2%)	249 (16.8%)	68 (19.5%)	117 (10.2%)	1835 (34.5%)	26 (30.6%)
	Some college	31 (9.1%)	443 (29.8%)	87 (25.0%)	336 (29.3%)	795 (14.9%)	12 (14.1%)
	Assoc/Tech degree	29 (8.5%)	165 (11.1%)	29 (8.3%)	114 (10.0%)	543 (10.2%)	7 (8.2%)
	HS grad or GED	9 (2.6%)	271 (18.2%)	47 (13.5%)	291 (25.4%)	391 (7.4%)	10 (11.8%)
	Less than HS grad	4 (1.2%)	206 (13.9%)	61 (17.5%)	214 (18.7%)	212 (4.0%)	6 (7.1%)
	Missing	1 (0.3%)	5 (0.3%)	0 (0%)	0 (0%)	0 (0%)	1 (1.2%)
**Insurance**							
	Government	34 (9.9%)	825 (55.5%)	147 (42.2%)	692 (60.4%)	764 (14.4%)	25 (29.4%)
	Commercial	289 (84.5%)	593 (39.9%)	178 (51.2%)	383 (33.5%)	4406 (82.8%)	54 (63.5%)
	Self-pay	86 (25.2%)	144 (9.7%)	47 (13.5%)	71 (6.2%)	1130 (21.2%)	15 (17.7%)
	Other	8 (2.3%)	31 (2.1%)	11 (3.2%)	34 (3.0%)	87 (1.6%)	2 (2.4%)
	**Income**						
	$0 - $49,999	52 (15.2%)	497 (33.4%)	130 (37.4%)	500 (43.7%)	1224 (23.0%)	27 (31.8%)
	$50,000 - $99,999	88 (25.7%)	251 (16.9%)	67 (19.3%)	117 (10.2%)	1576 (29.6%)	19 (22.4%)
	$100,000 - $149,999	61 (17.8%)	107 (7.2%)	39 (11.2%)	44 (3.8%)	982 (18.5%)	18 (21.2%)
	$150,000 - $199,999	45 (13.2%)	38 (2.6%)	14 (4.0%)	22 (1.9%)	533 (10.0%)	6 (7.1%)
	$200,000 +	64 (18.7%)	33 (2.2%)	16 (4.6%)	16 (1.4%)	518 (9.7%)	4 (4.7%)
	Missing	32 (9.4%)	561 (37.7%)	82 (23.6%)	446 (39.0%)	489 (9.2%)	11 (12.9%)
**Type of Labor**							
	Spontaneous, not augmented	69 (20.2%)	270 (18.2%)	67 (19.3%)	168 (14.7%)	953 (17.9%)	15 (17.7%)
	Spontaneous, augmented	164 (48.0%)	650 (43.7%)	130 (37.4%)	462 (40.4%)	2181 (41.0%)	32 (37.7%)
	Induced	81 (23.7%)	475 (31.9%)	131 (37.6%)	463 (40.4%)	1808 (34.0%)	33 (38.8%)
	Cesarean w/o labor or induction	28 (8.2%)	91 (6.1%)	20 (5.8%)	51 (4.5%)	379 (7.1%)	5 (5.9%)
**Mode of Delivery**							
	Vaginal	234 (68.4%)	1046 (70.3%)	264 (75.9%)	788 (68.8%)	3923 (73.7%)	55 (64.7%)
	Cesarean						
	Planned or scheduled	19 (5.6%)	60 (4.0%)	11 (3.2%)	32 (2.8%)	282 (5.3%)	3 (3.5%)
	Unscheduled, non-urgent	49 (14.3%)	188 (12.6%)	44 (12.6%)	181 (15.8%)	702 (13.2%)	21 (24.7%)
	Unscheduled, urgent	40 (11.7%)	193 (13.0%)	29 (8.3%)	144 (12.6%)	411 (7.7%)	6 (7.1%)
		Mean ± Std	Mean ± Std	Mean ± Std	Mean ± Std	Mean ± Std	Mean ± Std
**Age**		31.0 ± 4.7	24.8 ± 5.52	25.0 ± 6.2	23.3 ± 5.3	28.2 ± 5.2	27.9 ± 5.1
**BMI**		23.3 ± 4.0	26.7 ± 6.0	27.1 ± 6.9	29.1 ± 8.2	25.8 ± 5.8	27.2 ± 6.3
**Gestational Age at Delivery (weeks)**		38.9 ± 1.9	38.8 ± 2.0	38.6 ± 2.1	38.4 ± 2.5	38.9 ± 2.0	38.4 ± 2.6
**Percent Federal Poverty (%)**		590.9 ± 315.8	293.3 ± 263.2	337.4 ± 295.1	208.0 ± 245.0	488.3 ± 299.8	394.3 ± 287.1

The most common pre-pregnancy conditions were mental health conditions (14.4%, n = 1261), hematologic conditions (13.8%, n = 1206), neurological conditions (13.0%, n = 1136), and lung conditions (12.5%, n = 1091) (S2 Table 4 in S2 Appendix). Non-Hispanic Black women experienced the highest rates of lung and hematologic conditions, Hispanic women experienced the highest rates of kidney conditions, and multiracial women experienced the highest rates of cardiovascular conditions. Non-Hispanic white women experienced the highest rates of neurological, gastrointestinal, and mental health conditions.

Of all women, 22.8% (n = 1989) had co-occurring pre-pregnancy conditions, i.e., 2 or more conditions. Non-Hispanic white women had the highest rates of co-occurring pre-pregnancy conditions (24.9%, n = 1327), followed by multiracial women (22.7%, n = 79), and Non-Hispanic Black women (20.0%, n = 229). The most common co-occurring pre-pregnancy conditions were Mental & Neurological (3.7%, n = 322), Lung & Neurological (2.8%, n = 248), and Hematologic & Lung (2.5%, n = 216) (S2 Table 5 in S2 Appendix). Among non-Hispanic white women, the most common co-occurring pre-pregnancy conditions were Mental & Neurological (4.7%, n = 248), Lung & Neurological (2.9%, n = 153), and Hematologic & Lung (2.1%, n = 111). Among non-Hispanic Black women, the most common co-occurring pre-pregnancy conditions were Hematologic & Lung (4.4%, n = 50) and Cardiovascular & Hematologic (3.4%, n = 39).

Compared with non-Hispanic white women, the unadjusted risk for blood transfusion and severe preeclampsia was higher in non-Hispanic Black women. Statistical adjustment for age, sociodemographic information, insurance, and BMI eliminated the elevated risk of blood transfusion in non-Hispanic Black and Hispanic women and eliminated the elevated risk of severe preeclampsia for multiracial women. Statistical adjustment attenuated but did not eliminate the elevated risk of severe preeclampsia in non-Hispanic Black women. The adjusted relative risk (aRR) of severe preeclampsia was higher in non-Hispanic Black women (aRR, 1.22; CI, 1.06–1.41) compared to non-Hispanic white women (Table 2).

**Table 2:** Multilevel Poisson regression with outcome severe preeclampsia. Bolding indicates statistical significance at 95% confidence. RR, risk ratio; CI, confidence interval; AUC, area under the receive operating characteristics curve.

		Model 1, crude	Model 2, adjusted^1^	Model 3, condition types^2^	Model 4, combined effects^3^
		RR (95% CI)			
Race					
	Non-Hispanic White (reference)	1.00	1.00	1.00	1.00
	Non-Hispanic Black	**1.45 (1.29–1.63)**	**1.22 (1.06–1.41)**	1.15 (0.99–1.34)	1.16 (0.99–1.35)
	Hispanic	1.09 (0.92–1.28)	1.04 (0.87–1.23)	0.97 (0.82–1.14)	0.97 (0.82–1.15)
	Multiracial	**1.28 (1.02–1.60)**	1.09 (0.88–1.35)	1.06 (0.85–1.32)	1.06 (0.85–1.31)
	Asian	0.93 (0.62–1.37)	0.98 (0.67–1.43)	0.96 (0.67–1.38)	0.97 (0.67–1.38)
	Other	1.26 (0.81–1.96)	1.20 (0.78–1.86)	1.21 (0.76–1.91)	1.21 (0.77–1.91)
Pre-Pregnancy Conditions					
	Cardiovascular			**1.77 (1.61–1.96)**	**1.71 (1.54–1.89)**
	Kidney			**1.27 (1.03–1.56)**	**1.25 (1.01–1.53)**
	Hematologic			0.90 (0.77–1.04)	0.80 (0.59–1.08)
	Gastrointestinal			0.71 (0.48–1.05)	0.70 (0.47–1.05)
	Gynecological			0.84 (0.70–1.02)	0.85 (0.71–1.02)
Combined effects					
	Cardiovascular: Hematologic				1.26 (0.91–1.75)
Overall AUC 0.50 (0.48–0.52)		0.60 (0.57–0.63)	0.65 (0.63–0.68)	0.66 (0.63–0.69)

1 Adjusted for confounders and includes pre-pregnancy conditions and combined effects that are individually associated with postpartum readmission

2 Adjusted for confounders and includes pre-pregnancy conditions that are individually associated with severe preeclampsia

3 Adjusted for confounders and includes pre-pregnancy conditions and combined effects that are individually associated with severe preeclampsia

After adjusting for confounders, we controlled for pre-pregnancy condition types (Model 3) and their combined effects (Model 4). Controlling for pre-pregnancy condition types for the outcome of severe preeclampsia attenuated the risk of severe preeclampsia in non-Hispanic Black women (aRR 1.15, CI, 0.99–1.34) (Table 2).

Pre-pregnancy condition types were associated with adverse maternal outcomes (see Tables 2–4). Autoimmune conditions significantly increased risk for blood transfusion (aRR, 1.36; CI, 1.04–1.78) and postpartum readmission (aRR, 1.55; CI, 1.22–1.97). Hematologic conditions were associated with a significantly increased risk for blood transfusion (aRR, 1.42; CI, 1.23–1.64). Cardiovascular and kidney conditions were associated with a significantly increased risk for severe preeclampsia (aRR, 1.77; CI, 1.61–1.96, and aRR, 1.27; CI, 1.03–1.56 respectively). Accounting for combined effects did not significantly impact effect estimates.

**Table 3:** Multilevel Poisson regression with outcome blood transfusion. Bolding indicates statistical significance at 95% confidence. RR, risk ratio; CI, confidence interval; AUC, area under the receive operating characteristics curve.

		Model 1, crude	Model 2, adjusted^4^	Model 3, condition types^5^	Model 4, combined effects^6^
		RR (95% CI)			
Race					
	Non-Hispanic White (reference)	1.00	1.00	1.00	1.00
	Non-Hispanic Black	**1.29 (1.06–1.57)**	1.20 (0.97–1.50)	1.19 (0.95–1.50)	1.21 (0.96–1.52)
	Hispanic	**1.28 (1.07–1.54)**	1.17 (0.95–1.45)	1.19 (0.96–1.48)	1.19 (0.96–1.48)
	Multiracial	0.98 (0.59–1.62)	0.94 (0.57–1.52)	0.86 (0.54–1.36)	0.84 (0.53–1.32)
	Asian	0.86 (0.47–1.59)	0.85 (0.46–1.57)	0.88 (0.48–1.63)	0.88 (0.48–1.63)
	Other	1.25 (0.67–2.32)	1.21 (0.66–2.22)	1.17 (0.66–2.06)	1.18 (0.67–2.09)
Pre-Pregnancy Conditions					
	Cardiovascular			1.11 (0.88–1.39)	1.17 (0.92–1.48)
	Endocrine			1.25 (1.00–1.56)	**1.28 (1.02–1.60)**
	Kidney			1.28 (0.93–1.75)	1.27 (0.93–1.75)
	Hematologic			**1.42 (1.23–1.64)**	**1.42 (1.23–1.64)**
	Autoimmune			**1.36 (1.04–1.78)**	**1.36 (1.04–1.79)**
	Gynecological			0.69 (0.48–1.00)	0.73 (0.50–1.06)
Combined effects					
	Cardiovascular: Endocrine				0.83 (0.38–1.83)
	Cardiovascular: Gynecological				0.67 (0.22–2.02)
Overall AUC		0.56 (0.52–0.60)	0.55 (0.51–0.59)	0.60 (0.56–0.64)	0.58 (0.54–0.62)

4 Adjusted for confounders including maternal age, insurance, body mass index (BMI), income and education

5 Adjusted for confounders and includes pre-pregnancy conditions that are individually associated with blood transfusion

6 Adjusted for confounders and includes pre-pregnancy conditions and combined effects that are individually associated with blood transfusion

**Table 4:** Multilevel Poisson regression with outcome postpartum readmission. Bolding indicates statistical significance at 95% confidence. RR, risk ratio; CI, confidence interval; AUC, area under the receive operating characteristics curve.

		Model 1, crude	Model 2, adjusted^7^	Model 3, condition types^8^	Model 4, combined effects^9^
		RR (95% CI)			
Race					
	Non-Hispanic White (reference)	1.00	1.00	1.00	1.00
	Non-Hispanic Black	1.17 (0.95–1.45)	0.96 (0.75–1.21)	0.97 (0.76–1.24)	1.01 (0.79–1.29)
	Hispanic	1.18 (0.98–1.43)	1.05 (0.85–1.28)	1.08 (0.89–1.31)	1.07 (0.88–1.30)
	Multiracial	1.20 (0.87–1.66)	1.10 (0.79–1.53)	1.04 (0.73–1.48)	1.01 (0.70–1.46)
	Asian	0.30 (0.05–1.61)	0.32 (0.06–1.71)	0.29 (0.07–1.16)	0.29 (0.08–1.05)
	Other	1.42 (0.96–2.11)	1.36 (0.90–2.04)	1.42 (0.94–2.16)	1.43 (0.95–2.15)
Pre-Pregnancy Conditions					
	Cardiovascular			1.20 (1.00–1.45)	**1.33 (1.04–1.68)**
	Lung			1.10 (0.90–1.34)	1.26 (0.99–1.61)
	Endocrine			1.19 (0.97–1.45)	**1.33 (1.03–1.71)**
	Hematologic			1.14 (0.94–1.39)	**1.32 (1.02–1.71)**
	Autoimmune			**1.55 (1.22–1.97)**	**1.52 (1.19–1.94)**
	Neurological			1.09 (0.90–1.32)	1.25 (0.99–1.58)
	Mental			0.97 (0.80–1.19)	1.06 (0.85–1.31)
Combined effects					
	Cardiovascular: Neurological				0.84 (0.57–1.25)
	Cardiovascular: Endocrine				0.76 (0.52–1.11)
	Cardiovascular: Hematologic				0.89 (0.61–1.32)
	Lung: Neurological				0.73 (0.43–1.23)
	Lung: Endocrine				0.80 (0.48–1.33)
	Lung: Hematologic				0.84 (0.51–1.40)
	Neurological: Mental				0.79 (0.50–1.27)
	Endocrine: Hematologic				0.82 (0.47–1.43)
	Hematologic: Gynecological				0.38 (0.13–1.18)
Overall AUC		0.55 (0.51–0.59)	0.58 (0.54–0.62)	0.57 (0.53–0.61)	0.56 (0.52–0.60)

7 Adjusted for confounders including maternal age, insurance, body mass index (BMI), income and education

8 Adjusted for confounders and includes pre-pregnancy conditions that are individually associated with postpartum readmission

9 Adjusted for confounders and includes pre-pregnancy conditions and combined effects that are individually associated with postpartum readmission

Mediation analysis was conducted on all statistically significant condition types and combined effects for each adverse maternal outcome. There were no significant results at p-value < 0.05; however, cardiovascular conditions accounted for 36.6% of the association between non-Hispanic Black race/ethnicity and severe preeclampsia at p-value 0.07.

Finally, we compared the predictive performance metrics, specifically the area under the receiver operating characteristics curve (AUC), of our regression models to determine the value of different maternal characteristics in predicting the risk of experiencing each of the adverse maternal outcomes (S3 Appendix, Table 1). We found gains in overall AUC with the addition of condition types for the prediction of severe preeclampsia (AUC (95% CI); 0.60 (0.57–0.63) to 0.65 (0.63–0.68)). We computed AUC for each racial and ethnic subgroup to understand how the models performed across the subgroups. AUC varied significantly by race for each adverse maternal outcome. Model 3, which includes race, confounders, and condition types as features, had an AUC range of 0.54–0.72 for blood transfusion, 0.38–0.61 for postpartum readmission, and 0.40–0.71 for severe preeclampsia.

## Discussion

### Main findings

Our findings suggest accounting for pre-pregnancy condition types explained some of the association between non-Hispanic Black race/ethnicity and severe preeclampsia. We found that including pre-pregnancy condition types improved diagnostic ability (AUC) in predictive models for severe preeclampsia in the overall cohort. These results highlight the potential for risk prediction using pre-pregnancy conditions in a diverse, but low-risk population.

Consistent with previous studies, [4, 21, 43–45] we found that identification as a minority race and ethnicity was associated with a higher risk of adverse maternal outcomes among a cohort of nulliparous individuals. Adding to some studies that have examined the impact of specific co-occurring condition combined effects, [20, 46, 47] our study focused on exploring all potential pre-pregnancy condition type combined effects to understand their association with adverse maternal outcomes (specifically severe preeclampsia, postpartum readmission, and blood transfusion) and quantified the value of including combined effects in predictive models. No pre-pregnancy condition combined effects were significantly associated with adverse maternal outcomes.

We also quantified the association between individual pre-pregnancy condition type and adverse maternal outcomes. Each adverse maternal outcome was associated with different pre-pregnancy condition types, except the autoimmune condition type, which was associated with increased risk for both blood transfusion and postpartum readmission. It is well known that pregnancy is associated with changes in the maternal immune system, and specifically the postpartum period is associated with autoimmune disease flares [48].

Our work adds to previous studies on the use of prediction models to predict various adverse outcomes, which have ranged from using symptoms and signs, [17, 19] laboratory tests and biomarkers, [49, 50] and demographics and medical history [51, 52]. With the inclusion of pre-pregnancy condition types, our model yields an AUC of 0.65 for predicting severe preeclampsia. This is comparable to the AUC of previously published obstetric comorbidity indices predicting severe maternal morbidity, which includes severe preeclampsia (Bateman, et al. [18]: AUC, 0.65 and Easter, et al. [17]: AUC, 0.70 as reported by Leonard et al. [19]). The prediction models for severe preeclampsia improved with the addition of pre-pregnancy condition types. Together, our findings and prior studies suggest that maternal morbidity cannot be accurately predicted using medical conditions alone as these models cannot account for structural factors that contribute to health inequities.

### Clinical implications

Understanding the potential reasons for adverse maternal outcomes is an important pathway to understanding and reducing racial and ethnic disparities and high rates of maternal morbidity and mortality.

Our findings suggest that the presence of pre-pregnancy conditions and confounders may explain some of the observed association between non-Hispanic Black race/ethnicity and severe preeclampsia. In a prior study, Black race was associated with increased odds of pregnancy-induced hypertension after adjustment for preexisting conditions and demographic factors [53]. However, our study adjusts for a different set of preexisting conditions. The prevention and management of these pre-existing conditions, specifically cardiovascular conditions, before, during, and between pregnancies could be an important consideration to decrease racial and ethnic disparities in adverse maternal outcomes. Although the nuMoM2b cohort study collected data on a wide range of pre-pregnancy conditions, we could not explain a large proportion of the disparities in maternal morbidity in this cohort. Adverse maternal outcomes and pre-pregnancy conditions have been linked to the environmental context and social conditions in which people exist [25]. For example, a study by Lueth et al. (2022) found that a high allostatic load, as an estimate of chronic stress, was significantly associated with adverse pregnancy outcomes and partially mediated the association between self-reported race and adverse pregnancy outcomes in the nuMoM2b dataset [54]. A wider array of determinants (structural racism, socioeconomics, political context, etc.) should be evaluated to explain racial/ethnic disparities in adverse maternal outcomes and to develop comprehensive interventions to promote health equity. Future work may also benefit from the inclusion of additional data such as the severity and management of morbidity and multimorbidity during the preconception, interconception, and postpartum periods to determine whether differential management or access to healthcare systems reduces the observed disparities.

Our work suggests that information about pre-pregnancy conditions can be useful in improving the ability to risk-stratify individuals. Predictive modeling may be helpful in further exploring the complex relationships of co-occurring conditions. However, more research is necessary to inform best clinical practices for use of predictive models with a focus on mitigating unintended consequences and preventing the exacerbation of disparities [55].

### Strengths and limitations

Our study has several strengths. Using a large and comprehensive dataset, we evaluated the association between race and ethnicity, pre-pregnancy conditions, and adverse maternal outcomes in a cohort of nulliparous women in the United States. This dataset contains thoroughly collected data that goes beyond a typical electronic health record including health history and conditions, demographics, and survey questionnaires. Our condition type groupings allowed for larger sample sizes of conditions and more accurate estimates of risk ratios. In addition, the use of feature selection algorithms allowed for the exploration of combined effects to improve model performance for predicting adverse maternal outcomes.

Our study has several important limitations. First, the analysis was limited to the data collected in the nuMoM2b dataset, which only includes nulliparous, predominantly non-Hispanic white women (61.0%) who received regular prenatal care at academic medical centers beginning in their first trimester; thus, our findings may not be generalizable to different birthing populations. Second, the nuMoM2b dataset also does not indicate the severity or management of pre-pregnancy conditions. Third, our dataset only collected data up to 14 days postpartum, although many postpartum readmissions occur after this timespan [56]. Finally, adverse pregnancy outcomes are rare and occur infrequently in the nuMoM2b dataset, particularly as we stratified by racial subgroups and by multimorbidity. A larger sample size may be able to identify more clinically relevant associations between pre-pregnancy conditions and maternal outcomes that occur infrequently.

## Conclusion

In addition to describing associations between race and ethnicity, pre-pregnancy condition types and their combined effects, and adverse maternal outcomes, our findings indicate that pre-pregnancy conditions may partially explain the association between non-Hispanic Black race/ethnicity and severe preeclampsia, which may be further explained by a wider array of determinants. Additionally, data collected at an initial prenatal care visit has utility for predicting the risk of experiencing an adverse maternal outcome. Our study findings have important implications for the preconception and antepartum care of individuals with pre-existing conditions as adequately assessing a patient’s risk is essential to providing risk-appropriate and equitable care to prevent adverse maternal outcomes.

## Supporting information

S1 Appendix Exclusion protocol. (DOCX)

S2 Appendix Methods. (DOCX)

S3 Appendix Results. (DOCX)
