# Supplementary material for: Racial/ethnic differences in pre-pregnancy conditions and adverse maternal outcomes in the nuMoM2b cohort: A population-based cohort study
Source: PLoS One. 2024 Aug 12;19(8):e0306206. doi: 10.1371/journal.pone.0306206 (PMC11318875; doi:10.1371/journal.pone.0306206)
Supplement: S1 Appendix — (DOCX) [file pone.0306206.s001.docx]

S1 Appendix. Exclusion protocol

For this secondary data analysis, we chose to exclude women with pregnancy outcomes including fetal death before 20 weeks gestation, elective termination, indicated termination, unknown outcomes, and if the participant refused to release their pregnancy outcome. We also excluded women that delivered at gestational age < 22 weeks or > 43 weeks. We excluded women that had an incomplete labor, delivery, and postpartum form (form “CMA” in the nuMoM2b database) and those with an incomplete across visit medical conditions and medications form (form “VXX” in the nuMoM2b database).^19^ Finally, we excluded women without self-reported race/ethnicity status and those without mode of delivery recorded.
