# Supplementary material for: Racial/ethnic differences in pre-pregnancy conditions and adverse maternal outcomes in the nuMoM2b cohort: A population-based cohort study
Source: PLoS One. 2024 Aug 12;19(8):e0306206. doi: 10.1371/journal.pone.0306206 (PMC11318875; doi:10.1371/journal.pone.0306206)
Supplement: S3 Appendix — (DOCX) [file pone.0306206.s003.docx]

S3 Appendix. Results

|  | Model 0 | Model 1 | Model 2 | Model 3 | Model 4 |
| --- | --- | --- | --- | --- | --- |
|  | Intercept | Race | (1) & Confounders | (2) & Condition Types | (3) & Condition Type Combined Effects |
| Blood Transfusion, AUC (95% CI) | | | | | |
| Non-Hispanic White | 0.51 (0.46 - 0.56) | 0.50 (0.50 - 0.50) | 0.54 (0.49 - 0.59) | 0.57 (0.52 - 0.63) | 0.58 (0.53 - 0.64) |
| Hispanic | 0.48 (0.41 - 0.55) | 0.50 (0.50 - 0.50) | 0.48 (0.41 - 0.55) | 0.54 (0.47 - 0.62) | 0.49 (0.41 - 0.58) |
| Non-Hispanic Black | 0.46 (0.38 - 0.54) | 0.50 (0.50 - 0.50) | 0.53 (0.46 - 0.60) | 0.60 (0.52 - 0.68) | 0.59 (0.51 - 0.67) |
| Multiracial | 0.56 (0.32 - 0.80) | 0.50 (0.50 - 0.50) | 0.50 (0.30 - 0.70) | 0.72 (0.48 - 0.96) | 0.64 (0.40 - 0.88) |
| Asian | 0.35 (0.32 - 0.37) | 0.50 (0.50 - 0.50) | 0.44 (0.42 - 0.46) | 0.55 (0.30 - 0.79) | 0.40 (0.38 - 0.42) |
| Other | 0.60 (0.11 - 1.00) | 0.93 (0.90 - 0.97) | 0.86 (0.81 - 0.90) | 0.55 (0.06 - 1.00) | 0.50 (0.01 - 1.00) |
| *Overall* | *0.49 (0.46 - 0.53)* | *0.56 (0.52 - 0.60)* | *0.55 (0.51 - 0.59)* | *0.60 (0.56 - 0.64)* | *0.58 (0.54 - 0.62)* |
| Postpartum Readmission, AUC (95% CI) | | | | | |
| Non-Hispanic White | 0.48 (0.42 - 0.53) | 0.50 (0.50 - 0.50) | 0.54 (0.48 - 0.59) | 0.52 (0.47 - 0.57) | 0.53 (0.48 - 0.58) |
| Hispanic | 0.50 (0.41 - 0.59) | 0.50 (0.50 - 0.50) | 0.61 (0.54 - 0.69) | 0.61 (0.53 - 0.69) | 0.60 (0.51 - 0.68) |
| Non-Hispanic Black | 0.58 (0.50 - 0.67) | 0.50 (0.50 - 0.50) | 0.62 (0.52 - 0.71) | 0.58 (0.48 - 0.68) | 0.54 (0.44 - 0.64) |
| Multiracial | 0.64 (0.46 - 0.82) | 0.50 (0.50 - 0.50) | 0.53 (0.34 - 0.72) | 0.60 (0.42 - 0.78) | 0.54 (0.35 - 0.73) |
| Asian^[[1]](#footnote-1)^ | 0.70 (NA - NA) | 0.50 (NA - NA) | 0.50 (NA - NA) | 0.50 (NA - NA) | 0.50 (NA - NA) |
| Other | 0.64 (0.31 - 0.97) | 0.50 (0.50 - 0.50) | 0.52 (0.50 - 0.55) | 0.38 (0.05 - 0.71) | 0.41 (0.08 - 0.74) |
| *Overall* | *0.49 (0.45 - 0.53)* | *0.55 (0.51 - 0.59)* | *0.58 (0.54 - 0.62)* | *0.57 (0.53 - 0.61)* | *0.56 (0.52 - 0.60)* |
| Severe Preeclampsia, AUC (95% CI) | | | | | |
| Non-Hispanic White | 0.50 (0.47 - 0.54) | 0.50 (0.50 - 0.50) | 0.58 (0.55 - 0.62) | 0.62 (0.59 - 0.66) | 0.63 (0.60 - 0.67) |
| Hispanic | 0.52 (0.46 - 0.58) | 0.46 (0.41 - 0.50) | 0.55 (0.48 - 0.62) | 0.68 (0.61 - 0.75) | 0.69 (0.62 - 0.76) |
| Non-Hispanic Black | 0.49 (0.45 - 0.54) | 0.50 (0.50 - 0.50) | 0.51 (0.46 - 0.56) | 0.59 (0.54 - 0.64) | 0.60 (0.55 - 0.65) |
| Multiracial | 0.46 (0.38 - 0.54) | 0.50 (0.50 - 0.50) | 0.73 (0.63 - 0.82) | 0.71 (0.60 - 0.82) | 0.74 (0.64 - 0.85) |
| Asian | 0.42 (0.31 - 0.53) | 0.50 (0.50 - 0.50) | 0.62 (0.46 - 0.79) | 0.69 (0.51 - 0.86) | 0.69 (0.52 - 0.86) |
| Other | 0.40 (0.35 - 0.44) | 0.50 (0.50 - 0.50) | 0.81 (0.76 - 0.86) | 0.40 (0.15 - 0.65) | 0.40 (0.15 - 0.65) |
| *Overall* | *0.50 (0.48 - 0.52)* | *0.56 (0.53 - 0.58)* | *0.60 (0.57 - 0.63)* | *0.65 (0.63 - 0.68)* | *0.66 (0.63 - 0.69)* |

**S3 Table 1: AUC by race for each model and adverse maternal outcome.** AUC, area under the receive operating characteristics curve; CI, confidence interval.

1. The sample size of Asian study participants with postpartum readmission (N=1, 0.3%) was too small to compute 95% confidence intervals [↑](#footnote-ref-1)
